# Life-Threatening Irinotecan-Induced Toxicity in an Adult Patient with Alveolar Rhabdomyosarcoma: The Role of a UGT1A1 Polymorphism

**DOI:** 10.1155/2017/2683478

**Published:** 2017-09-26

**Authors:** Arnaud Jannin, Benjamin Hennart, Antoine Adenis, Bruno Chauffert, Nicolas Penel

**Affiliations:** ^1^Lille II University Medical School, Lille, France; ^2^CHU Lille, Service de Toxicologie et Génopathies, Lille, France; ^3^Medical Oncology Department, Oscar Lambret Cancer Centre, Lille, France; ^4^Medical Oncology Department, Amiens University Hospital, Amiens, France

## Abstract

Alveolar rhabdomyosarcoma (AR) in adult patients is an exceptional malignancy. Management of AR is based on (neo)adjuvant chemotherapy combining ifosfamide, vincristine, and actinomycin D and local curative-intent surgery/radiotherapy. In cases of relapsing AR, the combination of temozolomide/irinotecan is regarded as a possible option. Here we describe life-threatening long-lasting toxicity related to the 1st cycle of irinotecan-based chemotherapy in a 56-year-old woman suffering from locally advanced and metastatic head and neck AR. The patient experienced grade 4 vomiting and diarrheas resulting in acute functional renal failure, associated with grade 4 neutropenia complicated by severe septic shock. The hospital stay duration was 40 days. The analysis of the uridine diphosphate glucuronosyltransferase 1A1 (UGT1A1) gene revealed homozygous UGT1A1 ^⁎^28 polymorphism with an associated homozygous mutation c.-3275T>G; the latter is associated with a decrease of about 80% of UGT1A1 transcription explaining this irinotecan induced toxicity. Physician must be aware of the potential hematological (mainly neutropenia and infectious disease) and digestive (mainly diarrhea) toxicities caused by irinotecan and especially when the patient presents a UGT1A1 ^⁎^28 homozygous allele. UGT1A genotyping performed before initiating treatment is useful to anticipate severe toxic reaction to irinotecan and improve the benefit/risk ratio of its use.

## 1. Introduction

Alveolar rhabdomyosarcoma (AR) is a very rare malignancy. Adult cases of AR are exceptional [1]. AR management includes treatment with (neo) adjuvant chemotherapy, combining ifosfamide, vincristine, actinomycin D, and local curative intent surgery/radiotherapy [2]. In cases of relapsing AR, there is no consensus on second-line treatment; however, the combination of temozolomide and irinotecan has been considered a possible option [3].

In this case report, we describe life-threatening, long-lasting toxicity occurring after the first cycle of irinotecan-based chemotherapy. This case stresses the role of a uridine diphosphate glucuronosyltransferase 1A1* (UGT1A1)* polymorphism in the magnitude of observed toxicity.

## 2. Case Description

A 56-year-old Caucasian woman presented with locally advanced (meningeal and bone skull extension) and metastatic (massive cervical lymph nodes and diffuse vertebral involvement) head and neck AR. The patient experienced a complete response after 5 cycles of ifosfamide/vincristine and actinomycin D combination and conformational radiotherapy on the primary site (50.4 Gray). The first-line chemotherapy was well tolerated without dose reduction or severe toxicity. Unfortunately, twelve months later, the patient presented with a metastatic relapse with retroperitoneal lymph node and pancreatic metastases. Before starting treatment, hematological and biological parameters (including liver parameters) were within normal ranges. Second-line chemotherapy was administered. The 5-day treatment regimen consisted of temozolomide (125 mg/m^2^) administered per os daily and irinotecan (50 mg/m^2^) administered by IV daily. However, on the 4th day of treatment, the patient experienced grade 4 vomiting and grade 4 diarrhea, requiring a hospital stay. The diarrhea and vomiting led to dehydration and acute functional renal failure. On day 4, biological analyses revealed grade 4 neutropenia (228 neutrophils/mm^3^), grade 3 anemia (hemoglobin 6.9 g/l), grade 4 thrombocytopenia (10 652 platelets/mm^3^), and hepatic cytolysis (transaminases were 3 times the upper limit of normal). Severe stomatitis precluded oral alimentation, requiring a hospital stay and enteral alimentation for 13 days. On day 9, the patient experienced septic shock related to severe diverticulitis and ileitis, which required management in an intensive care unit and treatment with large spectrum antibiotherapy (piperacillin-tazobactam and amikacin). The neutropenia lasted 14 days, and the diarrhea lasted 18 days. The total duration of the hospital stay was 40 days. During the stay, she received 3 units of red blood cells and 5 units of platelets. Because of progressive disease, a third-line treatment with gemcitabine/dacarbazine is in progress.

In parallel, we hypothesized the presence of a predisposing pharmacogenetics condition. Our analysis of the* UGT1A1* gene revealed that the patient possessed a* UGT1A1* ^*∗*^*28* polymorphism. This polymorphism is frequently identified in the Caucasian population (0.387) [4]. Furthermore, genetic analysis revealed an associated homozygous mutation c.-3275T>G. The mutation associated with this polymorphism has been described to be responsible for a decrease in* UGT1A1 *transcription by approximately 80% [5].

## 3. Discussion

AR occurs rarely in adults and is associated with a poor prognosis [1]. There is no standardized treatment described for adults, and this is likely related to the limited number of studies in this group. The chemotherapy backbone for metastatic AR contains ifosfamide, vincristine, and actinomycin D (IVA protocol). After this first line of treatment and in the case of progression, temozolomide combined with irinotecan could be a therapeutic option [3].

Irinotecan is an anticancer agent widely used for advanced stage colorectal cancer. The activation and metabolism of this drug involve the sequential activation of SN-38 (7-ethyl-10-hydroxycamptothecin), the potent topoisomerase inhibitor, and detoxification of SN-38 to the pharmacologically inactive SN-38 glucuronide (SN-38G) by UGT1A1 [6]. The UGT1A1 enzyme is responsible for hepatic bilirubin glucuronidation. Reduced* UGT1A1* gene expression leads to Gilbert's syndrome, Crigler–Najjar syndrome [7], and irinotecan toxicity. More than 60 variants of* UGT1A1* have been described. Expression of* UGT1A1* is, in part, controlled by a polymorphic dinucleotide repeat sequence within the* UGT1A1* promoter TATA box, consisting of between five and eight copies of a TA repeat ([TA]nTAA). The (TA)_6_TAA allele is the most common (considered wild-type), and (TA)_7_TAA is the most frequently recorded variant allele (usually denoted by* UGT1A1* ^*∗*^*28*). The presence of seven or eight TA repeats in the* UGT1A1* promoter region leads to reduced glucuronidation, reduced SN-38G formation, and increased irinotecan-induced toxicity.

The frequency of the* UGT1A1* ^*∗*^*28* allele has been assessed worldwide. The* UGT1A1* ^*∗*^*28* allele is present in approximately one-third of Caucasians. This variant is a major predictive pharmacogenetic marker of severe toxicity during irinotecan-based chemotherapy [8]. Indeed, the association between drug toxicity and* UGT1A1* ^*∗*^*28* was analyzed in the Phase III TRIBE trial, which investigated the combination of bevacizumab-FOLFOXIRI versus bevacizumab-FOLFIRI. Among 443 evaluable patients, those carrying the* UGT1A1* ^*∗*^*28/* ^*∗*^*28* homozygous variant were at higher risk of developing grade 3/4 neutropenia compared with* 1* ^*∗*^*/28* and* 1* ^*∗*^*/1* carriers (59 versus 35%  *p* = 0.003) irrespective of the treatment arm [9]. Several studies utilizing the FOLFIRI protocol have suggested that the highest tolerated dose of irinotecan in patients with the* UGT1A1* ^*∗*^*28 *genotype was 130 mg/m^2^ [8, 10].

In the present case, the patient carries homozygous* UGT1A1* ^*∗*^*28* polymorphisms and NM_000463.2 (UGT1A1):* c.-3275T>G *mutations. The presence of both genotypes explains the long-lasting, severe hematological, and digestive toxicity. Our patient presented neither with jaundice nor isolated elevation of serum bilirubin levels, both of which might have suggested the presence of the UGT1A polymorphism.* UGT1A1 *genotyping performed prior to the initiation of irinotecan treatment could have allowed us to anticipate the development of this toxicity, but this genotyping is not a standard of care. However, the use of* UGT1A1* genotyping in routine practice is debated, especially in the setting of colorectal cancer. The 2016 ESMO guidelines suggest* UGT* genotyping in the following situations [11]: patients with a suspicion of UGT1A1 deficiency and patients receiving a dose of irinotecan > 180 mg/m^2^. Similarly, the National Comprehensive Cancer Network guidelines version 2.2016 states that irinotecan should be used with caution and at a decreased dose in patients with Gilbert syndrome. Nevertheless, routine testing of* UGT* SNPs is not recommended, and dose modifications according to genotyping are not a standard practice [12]. Recently, a note was added to the irinotecan FDA label suggesting an initial dose reduction for patients carrying homozygous* UGT1A1* ^*∗*^*28* alleles [13]. The French National Network of Pharmacogenetics group has recently suggested that* UGT1A1* genotyping is advisable for standard irinotecan doses and essential for intensified doses (>240 mg/m^2^) [14]. For doses between 180 and 230 mg/m^2^, this group recommends a 25% to 30% dose reduction in homozygous* UGT1A1* ^*∗*^*28* allele patients [14]. As recommended, our patient received a 50 mg/m^2^ dose of irinotecan per day from day 1 to day 5, so she received 250 mg/m^2^ in total [3]. According to the current recommendations of ESMO [11], all AR patients receiving the temozolomide/irinotecan in combination should receive UGT1A1 genotyping.

One reason for the nonimplementation of this genotyping test in clinical practice is its financial cost. An Italian study has precisely shown that the mean cost per patient was higher when genotyping was not implemented:  ^*∗*^*28/* ^*∗*^*28 *(€4.886) versus  ^*∗*^*1/* ^*∗*^*1 *(€812) (regression coefficient 1.79, 95% confident interval [1.31–2.28]; *p* < 0.001). This study also showed that, in Italy, the cost of toxicity management far exceeds the costs required to genotype all patients prior to initiating irinotecan treatment [15]. A prospective randomized European study is needed to determine if preemptive* UGT1A1 *genotyping could effectively reduce toxicity occurrence and related costs.

## 4. Conclusion

Following an IVA protocol in adults, refractory AR may require the use of irinotecan in combination with temozolomide. Clinicians must be extremely attentive to hematological (mainly neutropenia and infectious disease) and digestive (mainly diarrhea) toxicities when prescribing irinotecan, especially in patients with homozygous* UGT1A1* ^*∗*^*28 *alleles.* UGT1A1* genotyping performed before the initiation treatment could anticipate severe toxic reactions to irinotecan and improve the benefit/risk ratio of its use.
